# Influence of Porosity on the Mechanical Behavior during Uniaxial Compressive Testing on Voronoi-Based Open-Cell Aluminium Foam

**DOI:** 10.3390/ma12071041

**Published:** 2019-03-29

**Authors:** Varun Sharma, Nenad Grujovic, Fatima Zivic, Vukasin Slavkovic

**Affiliations:** Faculty of Engineering, University of Kragujevac, 34000 Kragujevac, Serbia; Varun.eu@gmail.com (V.S.); gruja@kg.ac.rs (N.G.); vukasinsl@gmail.com (V.S.)

**Keywords:** Voronoi tessellation, open-cell, porous structure, compressive load, stress, strain

## Abstract

We have studied an application of the Voronoi tessellation method in the modeling of open-cell aluminium foam under uniaxial compressive loading. The Voronoi code was merged with computer-aided design (CAD) for converting the polyhedral model into an irregular open-cell cellular structure to create porous samples for compression testing simulations. Numerical simulations of the uniaxial compression uniformly over the upper surface of the sample in the *z*-axis direction at a constant 20 N load was realised. Samples with three different porosities (30%, 60% and 80%) were studied. A nonlinear elasto-plastic material model with perfect plasticity, without hardening, based on the von Mises yield criterion was applied below 10% strain. Corresponding stress–strain curves were observed and the influence of porosity on deformation mechanism was discussed. Samples with higher porosity exhibited significantly higher normal stress under the same load, and increased stress plateaus. An increase of porosity produced an increase of both compressive and tensile stresses and struts exhibited complex stress fields. Voronoi-based modeling was in accordance with experimental results in the literature in the case of the quasi-static condition and linear elastic region (below 1% strain). Further study is necessary to enable the simulation of real dynamic behaviour under all deformation regimes by using the Voronoi tessellation method.

## 1. Introduction

Aluminium foams have became the subject of research interest in recent years due to their unique physical and mechanical properties when compared to fully dense metallic materials [1,2,3,4,5,6]. The lightweight structure and properties related to crash behaviour has made it a promising candidate for applications that require good energy and vibration absorption properties during impact [7,8]. The automotive industry has a massive use of aluminum foams in many major components. However, open-cell foams have a complex natural geometry and their mechanical behaviour is still under investigation, especially in the form of numerical modeling. Application of computational methods for the design and determination of mechanical properties is not an easy task due to the complex microstructure and characteristics of the material itself [9].

Aluminium foams belonging to the metal matrix syntactic foams (MMSFs) offer various advantages [10] for applications in different industrial sectors, such as automotive [11], aerospace [12], military, and chemical industries [13], due to excellent properties, such as energy absorption abilities, thermal insulation capabilities, and electromagnetic shielding effect [14], besides being a lightweight material. Metallic panels and tubes are used to fabricate aluminium foam sandwiches (AFS) [15] and foam-filled tube structures (FFTSs) [16]. Accordingly, the properties of aluminium foams related to bending [17] and impact resistance [18] have been significantly improved. Reinforced metal wires and meshes have been used to prepare reinforced aluminium foams.

In general, metal matrix syntactic foams (MMSFs) are multiphase materials, consisting of a metallic matrix and a set of hollow inclusions [19]. Metallic foams have a combination of properties that make them attractive in a number of engineering applications. In structural sandwich panels, they offer a low weight and honeycomb structure that exhibit good properties under compressive loading. Recently developed processing techniques allow for manufacturing at a relatively low cost. Metal foams can withstand large displacements, shortening the whole structure (up to 60%), thus enabling fabrication of efficient energy absorption devices. Open-cell metallic foams are composed of an excellent heat dissipation structure, which gives them their high thermal conductivity, their high internal surface area, and the connectivity of the voids, which allows a cooling gas to pass through.

Theoretically, MMSFs can be produced from any kind of metals, but their matrix is usually a grade of lightweight alloy (Al, Mg, Fe, Zn, Ti) [20]. Melt forming (MF), gas injection (GI), powder metallurgy (PM), and infiltration casting (IC) have been exploited for the manufacture of both closed-cell and open-cell aluminum foams [10]. However, further research is needed regarding several possible applications related to improvements of the mechanical properties. For example, adding alloying elements, such as Mn and Sc, can enhance the compressive strength of aluminium foams. Adding microparticles, such as nano-SiC [21], or fly ash [4] and carbon nanotubes [22], have been investigated for reinforced aluminium foams with higher yield strength than pure aluminium foams. In order to improve mechanical properties, high-strength materials have been introduced into, or onto, aluminium foams. Orbulov [23] introduced iron hollow spheres into the metal matrix and fabricated syntactic foams, with significantly improved compressive strength, being the most common loading mode for aluminium foam [20]. Compression tests for foams are the only standardized tests performed on MMSFs [24], which include characteristic strength values (compressive strength, plateau stress, quasi-elastic gradient, elastic gradient, compressive offset stress, compressive proof strength) and the characteristic strain values (e.g., the deformation at the plateau end). Beside this, some authors suggest testing in all basic loading modes, including bending, tension and even impact [17,18,25,26,27,28].

Numerical methods and analysis are powerful tools in material design, characterisation and prediction of properties during function. Their application for porous structures has begun in the last decade but it is still in development. Several methods have been investigated for the analysis and modelling of open-cell and closed-cell aluminium foams [29,30]. Finite element modelling (FEM) can indicate the relationship between deformation mechanisms and relative foam density [6]. Multi-scale modelling was applied to design foam components with complex and irregular shapes [31]. An FEM-based method for high velocities has been used to model the velocity prototype of impact collisions for cellular materials, whereas the model can be expanded to the total deformation of the body with an appropriate accuracy in relation to analytical models, or to determine the degree of deformation of heterogenic materials exposed to impact under low and normal velocities [32]. Numerical modelling based on the elastic-plastic deformation behaviour of samples of open-cell aluminium foams revealed that the plateau value and energy absorption increase with decreasing void size and increasing density [33].

Recently, the Voronoi tessellation method was used to create a 3D foam model. Some studies showed that simulations of three-dimensional Voronoi tessellation models can be used to understand the direct relationship between mechanical properties and the regularity parameter [34,35]. Schladitz et al. [36] reviewed the theoretical background for 3D imaging by using microcomputed tomography, in particular estimating the intrinsic volumes and their densities from discretized data and models for random spatial tessellations. Laguerre tesselations, coupled with Voronoi tessellations, has been used to model the foam structures with good results [37,38]. Analysis of heterogeneous materials containing a dispersion of ellipsoidal inclusions or voids in the matrix was done by using a three-dimensional Voronoi cell finite element model [39]. Experimental studies pertaining to compressive properties of open-cell Al foams have been realised from different aspects: influence of strain rate on deformation mechanisms [40], influence of processing routes and strain rate on compressive response [41], and high strain rate compressive behaviour [42]. These experimentally obtained results represent a good foundation for numerical modeling simulations.

We have used a Voronoi code merged with computer-aided design (CAD) for converting a polyhedral model into an irregular open-cell cellular structure. The generated structure was further used in finite element analysis (FEA) under uniaxial uniform compressive loading, with three different levels of porosity: 30%, 60% and 80% porosity. Corresponding stress–strain curves were observed along with the influence of porosity on deformation mechanism.

## 2. Methodology and Assumptions

### 2.1. Voronoi Tessellation Method (VTM)-Based Open-Cell Model

**Figure 1:**
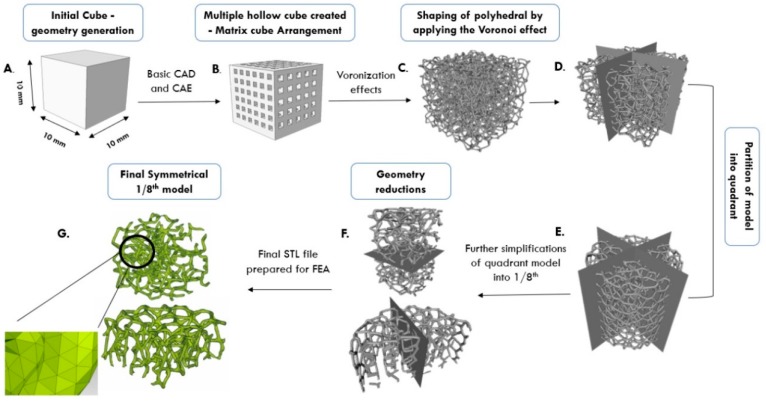
Design schematic of open-cell metallic foam using the Voronoi tessellation method. (CAD—Computer-aided design; CAE—Computer-aided engineering; STL—“stereolithography” file format; FEA—Finite Element Analysis).

The Voronoi tessellation method (VTM) was used to model the irregular open-cell structure, similar to the real geometry of an insulation material structure made of Al-based alloys. This method enables different variations of void arrangement, cell wall thickness, porosity level, and density. VTM is a technique that allows the generation of points or seeds in space, which are piled into clusters to further form Voronoi cells. They usually have polyhedral shapes and can conveniently follow assigned points or seeds. Usually, voids are generated in a zigzag form and connected together in a cluster. Initially, we tested hollow sphere structures generated using the commercial code Femap with NxNastran software (Version 10.3) by using variations of different sizes and circular shapes of voids, strut thicknesses and number of cells. However, this method does not allow generation of random irregular structures. Accordingly, we have used the VTM approach by using Voronoi polyhedra, either as individuals or as a group of cells. VTM-based modeling allowed for variations of strut length and thickness, and polyhedral diameter. Schematic representation of the steps in the generation of irregular open-cell structure by VTM is shown in Figure 1.

Starting solid cube 10 × 10 × 10 mm^3^ with internal cubes was used and the Voronoi tessellation effect was applied to perform the conversion of the cubes into polyhedrons. This transition can be done either by executing MATLAB^®^ or Octave source code, or by using the inbuilt software algorithm. This conversion here was accomplished by using open source Voronoi code. Voronoi coding process generated a file in PLY or STL (Polygon/Stereo lithography binary) format, which may further be passed to the finite element method (FEM) software for analysis. However, the processing of STL files using FEM software is time and resource intensive due to the raw unstructured triangulated surfaces. In order to adjust the STL format to suit further finite element analysis (FEA), MeshLab software (Version 2016.12) was employed. Even after that processing, the structure was too large, with a large number of elements (nodes and polyhedral voids) for further analysis in Femap with NxNastran software (Version 10.3). Partitioning into smaller volumes was performed using Femap with NxNastran software, as shown in Figure 1. Through implementation of representative volume method, with 1/8^th^ of the initial model (at least 24 cells were comprised within this representative volume), the whole structural response was numerically computed using three different levels of porosity: 30%, 60% and 80% porosity. All the voids were arranged linearly within the outer enclosed cube. Input parameters of numerical computation are given in Table 1.

### 2.2. Meshing

In general, a structure design such as open-cell foam, is not influenced by the mesh size due to chaotic behaviour of material itself. However, in order to understand the influence of voids, struts and the level of porosity, it is important to use an appropriate mesh size. After several mesh refinements and evaluating the resulting discontinuities in our numerical simulation models, we used three different models (global mesh size of 0.02) consisting of more than 100,000 elements, and Jacobian 0.6, as given in Table 2. Solid 3D quadratic tetrahedron meshes with tetrahedral 10 node elements were assigned uniformly to the whole geometry. These mesh sizes had final element contours that related well to real models of metallic foams.

### 2.3. Nonlinear Modelling of Open-Cell Aluminium Foam

Modeling started by importing the Voronoi STL file into the FEA solver for the geometry clean-up process in order to extract the silver faces generated in the geometry due to numerical inaccuracies in solid modelling operations [17]. Relatively small sizes of created silver faces can cause numerical instabilities in iterations. A Cartesian coordinate system was used for the material orientation of elements. Isotropic material behaviour was observed with material properties given in Table 3. Aluminium foam was used as the case example due to its broad application in various industries [1].

**Figure 2:**
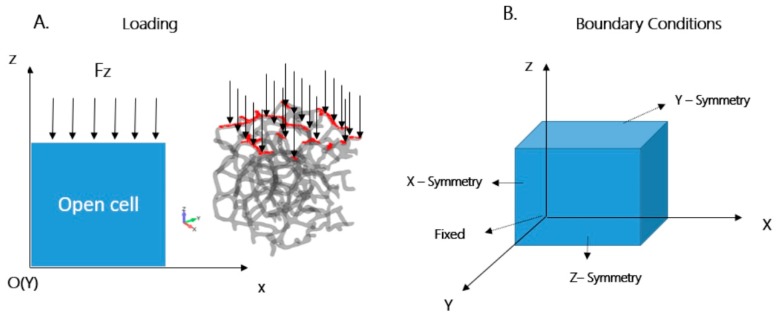
Schematic representation of boundary conditions: (**A**) uniform compressive loading of 20 N, only in the *z*-axis direction, over the entire upper surface of the sample; (**B**) symmetrical boundary conditions applied in the respective directions.

A uniaxial compressive loading of 20 N was applied along the *z*-axis direction, uniformly over the upper surface of the structure, as shown in Figure 2A. The load of 20 N was selected according to applications where this Al-based foam can be used, as per literature data pertaining to experimental results where compressive loading was investigated [35,40,41] and to avoid a large strain as in References [33,42]. The maximum load of 20 N was setup as a constant, and the loads versus time function was applied in Femap software.

We used strain control (strain dependent threshold) for automatic load stepping in order to prevent the calculations for the large strain because the applied numerical model is valid below 10% strain. Accordingly, calculations in the numerical simulation analysis were run until the software automatically determined the moment when the maximum allowed strain was reached; that is, when the longitudinal strain reached its limit value. When the sample was deformed above its limit value, further calculations were automatically stopped and the graph was plotted. Numerical values of von Mises stress, generated by the software, were observed in three different directions (*x*, *y*, *z*), along shear planes (*xy*, *yz*, *zx*), as well as the solid maximum stress, shear stress and mean stress, for three different samples (30%, 60%, 80% porosity), as the function of strain.

Four rotational and translational degrees of freedom were applied, as shown in Table 4: (a) along the *x*-axis direction, only translational in the *x*-axis direction and rotation in *y*- and *z*-axis directions; (b) along the *y*-axis direction, only translational in the *y*-axis direction, and rotation in *x*- and *z*-axis directions; (c) along *z*-axis direction, only translational in the *z*-axis direction, and rotation in *x*- and *y*-axis directions. One node in the lower part of the model was selected and fixed (as shown in Figure 2B), in order to simulate boundary conditions with stationary path.

For compressive tests of the open-cell foam, a nonlinear elasto-plastic material model following the von Mises yield criterion was applied. Aluminium exhibits a ductile nature and von Mises yield criterion that can also be used for shear simulation. The material acts in a linear elastic manner until the stress exceeds the yield stress (Equation (3)). Von Mises criterion states that the yielding begins when the elastic stress (or second invariant of deviatoric stress, $J_{2}^{\prime }$) exceeds a critical value. In simple uniaxial tension or compression, the critical values are related to the yield stress (*Y*), i.e.,:

(1) $$\sqrt{3}\;(J_{2}^{\prime }{)}^{½}\;>\;Y$$

where

(2) $$J_{2}^{\prime }=\frac{1}{2}\sigma_{ij}^{\prime }=\sqrt{\frac{1}{6}}\left[{\left(\sigma_{x}-\sigma_{y}\right)}^{2}+{\left(\sigma_{y}-\sigma_{z}\right)}^{2}+{\left(\sigma_{z}-\sigma_{x}\right)}^{2}\right]$$

Equation (2) can be expressed in terms of a general stress state as:

(3) $$\sigma_{VonMises}=\sqrt{\frac{1}{2}}\left[{\left(\sigma_{x}-\sigma_{y}\right)}^{2}+{\left(\sigma_{y}-\sigma_{z}\right)}^{2}+{\left(-\sigma_{x}\right)}^{2}\right]+3\left(\tau_{xy}^{2}+\tau_{yz}^{2}+\tau_{zx}^{2}\right)$$

By combining Equations (2) and (3):

(4) $$\tau \;=\;\frac{Y}{\sqrt{3}}$$

where *Y* is the yield stress of the material, i.e., yield stress in pure shear is 1/$\sqrt{3}$ times the yield stress in simple uniaxial tension and compression. A full Newton–Raphson iteration method was employed for numerical computing in the finite element software.

## 3. Results and Discussion

The Voronoi tessellation method (VTM) was used to model irregular open-cell aluminium foam and study the compression response in the elastic and plastic deformation regimes and the influence of different porosities on compressive behaviour. We used strain control in the numerical calculation to limit the loading, below a 10% strain, with the maximum load of 20 N in all calculations, as appropriate for low strain compression. Von Mises stresses were observed in three different directions (*x*, *y*, *z*), along shear planes (*xy*, *yz*, *zx*), as well as solid maximum stress, shear stress and mean stress, for three different porosities (30%, 60%, 80%), as the function of strain.

**Figure 3:**
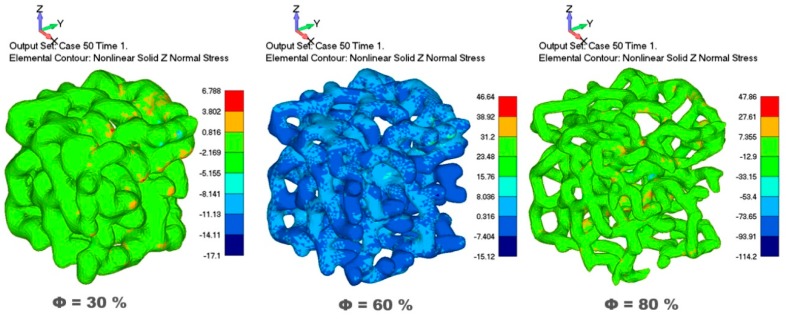
Von Mises stress contours in the test direction for three different porosities under uniaxial uniformly applied load.

Comparison of calculated normal stresses in the cases of 30%, 60% and 80% porosities showed that models with higher porosity exhibited significantly higher normal stress, as shown in Figure 3. Models with higher porosity had larger voids, thus allowing significantly higher shortening of the sample and struts displacements under the same compressive loading. With an increase of porosity, both compressive stresses and tensile stresses increased, as can be seen in Figure 3. Large shortening of the sample during compression, enabled by empty space of voids, such as in case of 80% porosity, influenced significant displacements, bending and extension of the struts. Very high values of compressive stress can be observed in some strut zones, as shown in the Von Mises stress contour in Figure 3 (red color in cases of 30% and 80% porosity and light blue color in case of 60% porosity).

Our simulation results are in accordance with other authors who experimentally showed that an increase in porosity resulted in a decrease in the foam strength [6,33,40,41]. Our numerical simulations were done by using a constant maximum load, rapidly achieved without progressive loading. Samples with higher porosity had more empty voids, thus having lower total contact surface subjected to loading in comparison to less porous samples. Stress is defined as the force across an area, and accordingly, an increase of porosity decreases the contact surface, thus influencing a more rapid increase of stress, and reaching of yield stress, as well as ultimate stress due to decrease in the foam strength. Zhou et al. [40] progressively loaded porous samples of Al-based foam and showed that a major deformation mechanism was the plastic bending of struts. The von Mises stress contour in Figure 3 exhibits different stress levels throughout the struts, whereas distinct zones with highly increased stress are clearly visible, especially in case of 80% porosity, indicating zones with extensive plastic bending, as suggested by Zhou et al. [40]. Figure 3 shows the simultaneous presence of tensile stress in some strut zones, thus indicating complex stress fields. Plastic bending, stretching and buckling of individual struts was also suggested by Shunmugasamy and Mansoor [41]. They experimentally produced porous Al foam via two different routes and samples with three levels of porosities (7%, 29% and 42% porosity) were subjected to compressive loading over a range of strain rates, also including high strain. Their studies focused on the investigation of the strain rate sensitivity of aluminum foam by using different strain rates and loading, whereas we used only one load and low strain. Their samples showed different deformation behaviour depending on the range of loads: linear elastic deformation at small stress levels, and plastic yielding and large strain for higher stresses. They proved that hardening occurred with strain increases. In the case of low strain rates [40,41], quasi-static compressive stress–strain curves are comparable with our simulation results since our numerical calculations were realised under the low strain range (below 10% strain). Sotomayor et al. [35] suggested that interactions among the struts had started around 10% strain, thus indicating that for our simulation model, we can neglect these interactions. We modeled the material structure as a material with perfect plasticity without hardening and based on the von Mises yield criterion.

**Figure 4:**
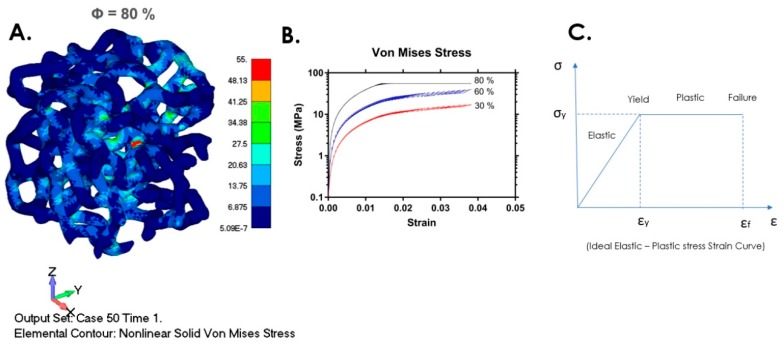
(**A**) Von Mises stress contour, sample with 80% porosity; (**B**) stress–strain curve, log form; (**C**) ideal elastic–plastic stress–strain curve.

The von Mises stress contour in case of 80% porosity and corresponding stress–strain curves are shown in Figure 4. Struts are subjected to the complex stress field and the resulting strain is the consequence of the simultaneous acting of normal stress and shear stress caused by compressive loading. Our results showed that shear stress has a significant role in the failure mechanism of the struts. In the cases of 60% and 80% porosity, almost all struts were subjected to the complex coupled effects of compressive and tensile stresses that acted within different regions of one strut.

**Figure 5:**
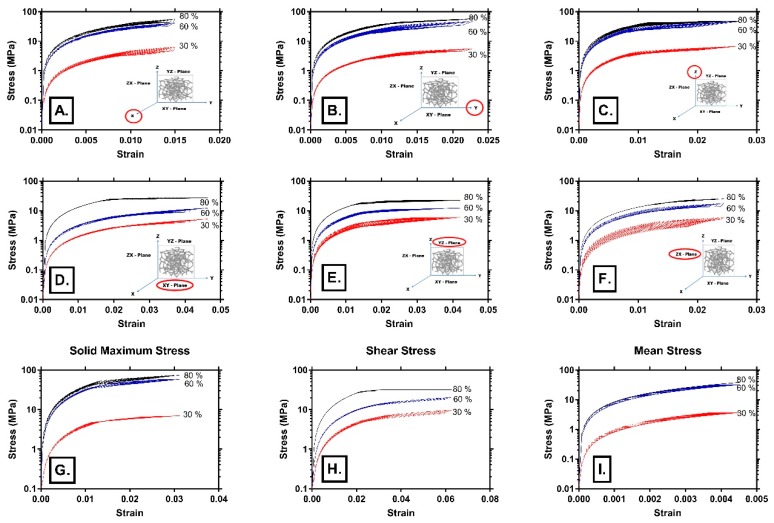
Stress–strain curves for three different porosities (30, 60, 80%): (**A**–**C**) stresses in *x*-, *y*- and *z*-axis directions as a function of strain, respectively; (**D**–**F**) shear stresses in *xy*, *yz* and *zx* directions as a function of strain, respectively; (**G**–**I**) maximum principal stress, maximum shear stress, and maximum mean stress, as a function of strain, respectively.

Von Mises stresses in three different directions (*x*, *y*, *z*), along shear planes (*xy*, *yz*, *zx*), and solid maximum stress, shear stress and mean stress, for three different porosities (30%, 60%, 80%), as the function of strain are shown in Figure 5. Samples with a lower porosity (30%) exhibited a significantly lower level of stress. It can be seen that the increase of porosity (30%, 60%, 80%) resulted in an increase of plateau stress. Similar to our results, Shunmugasamy and Mansoor [41] showed that the increase of porosity (7%, 9% and 42% in their tests) increased plateau stress.

Deshpande and Fleck [42] showed that, in the case of two types of aluminium foams (close-cell Alulight foam and tetrakaidecahedron shaped open-cell Duocel foam), dynamic behaviour under high strain is not different to their quasi-static behaviour. Their tests showed that below the strain rate of 5000 s^−1^, plateau stress was not sensitive to strain rate. The applied model in our simulation tests excluded hardening, as can be seen in Figure 4B and Figure 5, and the stress–strain curves are in accordance with the experimental results [6,40,41,42] under two regimes: linear elastic and plateau regimes of their tests. Almost flat stress plateaus can be seen in Figure 4B and Figure 5, clearly indicating a lack of hardening, since it was not encountered by this VTM-based model. Our model can be valid in some cases of quasi-static condition, according to experimental results such as in [41]. However, for real dynamic behaviour beyond the limit load that we setup in this model, additional model adjustments were necessary, such as multiscale modeling.

In our model, the nominal stress plateau was sensitive to the change of porosity up to some limit. It can be seen in Figure 5A that for axial stress in the loading *z*-axis direction, stress plateaus in the cases of 60% and 80% were almost identical. From some level of high porosity, defects in the microstructure of cells and local yielding probably occurred rapidly upon loading. This was also supported by the shear stress curves in Figure 5B,C, indicating that it had a more prominent role in the deformation mechanism of the highly porous structure (60% and 80% porosity). It is probable that bending and buckling of struts along with slip bands formation governs the deformation in such case, which is also suggested in [41]. Deshpande and Fleck [42] also suggested that below some strain rate, plateau stress became insensitive to strain.

Some authors used a tetrakaidecahedron shape as the unit cell in finite element models of porous structures [43] or an elongated tetrakaidecahedron [44]. They also obtained good agreements with experimental data in the case when the geometry of struts was well assigned to the Kelvin cells [43,45]. Jang et al. [43] studied aluminium foam (relative densities of about 8%) using experimental tests and developed numerical models, based on Kelvin cells, by using those experimental data. The shape of stress–strain curves within a linear elastic region (also below 10% strain in their tests) were comparable to the stress–strain curves in our numerical simulation. Similar to our numerical model, their model using Kelvin cells showed good agreement with the experimental compressive response, up to the limit stress. Beyond that limit, they used finite size 3D domains to evaluate the compressive response. Sullivan et al. [44] studied the influence of the additional shape parameter (elongated tetrakaidecahedron) in order to develop a theoretical model for non-isotropic mechanical behaviour of open-cell foam. This model required physical and mechanical measurements pertaining to the foam microstructure prior to the application of developed equations. Other authors also suggested multiscale modeling in order to properly fit models, also by including microstructural heterogeneities of the foam [6].

For the linear elastic region (below 1% strain), our numerical model, based on the Voronoi tessellation method, showed good agreement with experimental results that can be found in the literature. The advantage of the Voronoi tessellations is that, once the code is applied, it is easy to change influential parameters (voids arrangement, strut thickness, porosity level, density) and observe their effects on the mechanical behaviour. Some issues with Voronoi-based modeling are related to the timely refinement of mesh, distorted surface, coon curve and edges skewness that would need further study by using optimisation methods.

## 4. Conclusions

The Voronoi tessellation method (VTM) was used as part of an investigation for the modeling of irregular open-cell structures. Voronoi polyhedrons were used to model aluminium foam under uniaxial uniform compressive loading. The starting solid cube unit cell was subjected to several computational processing, including Voronoi coding, to form Voronoi unit cells. The resulting structure was optimised by partitioning it into a representative volume that was subjected to a compression test simulation. Three porous samples (30%, 60% and 80% porosity) were subjected to uniaxial compressive testing at 20 N along the *z*-axis direction, uniformly over the upper surface. The influence of the porosity on the compression response of open-cell structure was studied in the elastic and plastic deformation regimes. Material structure was modeled as a perfectly plastic material without hardening and based on the von Mises yield criterion. A nonlinear elasto-plastic material model for open-cell aluminium foam was used. A load versus time function, with strain control, was applied in Femap with NxNastran software. Numerical calculations were automatically stopped when the longitudinal strain reached its limit value, thus keeping the simulation below 10% strain. Von Mises stresses, maximum principal stresses and mean stresses as a function of strain were studied. The comparison of normal stresses in the cases of 30%, 60% and 80% porosity showed that samples with a higher porosity exhibited significantly higher normal stress under the same load. With an increase of porosity, both compressive stresses and tensile stresses increased. The increase of porosity (30%, 60%, 80%) resulted in an increase of plateau stress. The nominal stress plateau was sensitive to the change of porosity up to some limit, but stress plateaus in cases of 60% and 80% were almost identical. The VTM-based model studied in this paper can be valid in some cases of quasi-static conditions and for a linear elastic region (below 1% strain). For the simulation of real dynamic behaviour beyond the limit load that was set up in this model, additional model adjustments are necessary, such as multiscale modeling or to include microstructural properties of struts. Also, further study is needed regarding aspects of meshing optimisation.

## Figures and Tables

**Table 1:** Input parameters of numerical computation for Voronoi tessellation method based open-cell foam.

Porosity	Internal Matrix Size Given for Voronoi Code (mm)	Strut Length (mm)	Void Diameter (mm)
30%	0.6–0.7	1–1.5	≈0.5
60%	0.4–0.5	0.75–1	≈1.0
80%	0.25–30	0.5–0.75	≈1.5

**Table 2:** Mesh sizes in finite element analysis, for three different porosity levels.

Porosity	Number of Tetrahedral 10 Node Elements
30%	152364
60%	177484
80%	128673

**Table 3:** Material properties of aluminium used as input parameters for numerical modeling of open-cell aluminium foam [17].

Density (Tonne/mm^3^)	Young Modulus of Elasticity (MPa)	Poisson Ratio	Yield Stress (MPa)
2.7 × 10^−9^	68200	0.3	55

**Table 4:** Degrees of freedom at modelling of open-cell aluminium foam.

Cartesian Coordinate Axes	Degree of Freedom					
	Translational in *x* Directions	Translational in *y* Directions	Translational in *z* Directions	Rotational in *x* Directions	Rotational in *y* Directions	Rotational in *z* Directions
*x*–Symmetry	✓				✓	✓
*y*–Symmetry		✓		✓		✓
*z*–Symmetry			✓	✓	✓	
Fixed Node	✓	✓	✓	✓	✓	✓
